# Impacts of future sea level change on Greenland from community knowledge, coastal mapping, and glacial isostatic adjustment models

**DOI:** 10.1073/pnas.2528615123

**Published:** 2026-06-01

**Authors:** Kirsty J. Tinto, Jacqueline Austermann, Robin E. Bell, David Blockley, Casey E. Brayton, Diana Krawczyk, Lauren Lewright, Andrew J. Lloyd, Frank O. Nitsche, Guy J. G. Paxman, David F. Porter, Aqqaluk Sørensen, Margie Turrin, Karl Zinglersen

**Affiliations:** ^a^ Marine and Polar Geophysics Division, Lamont-Doherty Earth Observatory, Columbia University, Palisades, NY 10964; ^b^ Seismology, Geology and Tectonophysics Division, Lamont-Doherty Earth Observatory, Columbia University, Palisades, NY 10964; ^c^ Department of Earth and Environmental Sciences, Columbia University, New York, NY 10027; ^d^ Department of Environment and Minerals, Greenland Institute of Natural Resources, Nuuk 3900, Greenland; ^e^ Greenland Climate Research Centre, Greenland Institute of Natural Resources, Nuuk 3900, Greenland; ^f^ Department of Geography, Durham University, Durham DH1 3LE, United Kingdom

**Keywords:** Greenland, GIA, bathymetry, sea level, community

## Abstract

Making predictions of global change relevant to individual communities is a critical challenge, especially where local impacts diverge from global predictions. While sea level is rising globally, in Greenland, sea level is predicted to fall over the coming century due to land rebound and sea surface lowering, consequences of glacial isostatic adjustment from both past and future ice loss. Here we present results of a US and Greenland scientific partnership, working with the community of Aasiaat, West Greenland, to identify the impacts of future sea level change where sea level is projected to fall 0.41 to 0.93 m by 2100 CE. Through collaboratively designed, conducted, and interpreted multibeam bathymetry surveys combined with targeted glacial isostatic adjustment models, we identify areas that will be most impacted by sea level fall. We identify two shallow channels where disruption to navigation and nutrient cycles will occur when water level falls beneath critical depth thresholds. To make these predictions useful we established communication pathways to share findings effectively across a broad array of interest groups by engaging with community members, government officials, and students. This multipronged approach of collaboratively designed observations, locally targeted impact modeling and integrated formal and informal communication can be expanded to coastal communities facing environmental challenges around the world.

## Significance

While global mean sea level is predicted to rise, the magnitude and direction of sea level change varies from place to place. Impact studies and adaptation strategies must be local, appropriately representing this predicted variability, local landscapes, and societal factors. This study presents new bathymetry and marine habitat mapping around Aasiaat, West Greenland through surveys that were designed, conducted, and interpreted in collaboration with local communities. As sea level here is predicted to fall, our results highlight locations vulnerable to lower sea level of significance to residents. Ongoing community engagement throughout the survey design and execution yielded usable impact analysis and new language to describe environmental change.

The Greenland Ice Sheet contributes 1.9 Gt/y of fresh water to the global ocean, accounting for ~14% of global sea level rise observed between 1992 CE and 2020 CE (1). This mass loss is projected to increase over the coming century, with consequences for coastal communities around the globe (2). While global mean sea level is projected to rise by 0.28 to 1.01 m [by 2100 CE, relative to 1995 CE to 2014 CE (2)], average local sea level around Greenland is projected to fall by 0.9 to 2.5 m relative to 2017 CE (3). This local sea level drop is a result of glacial isostatic adjustment (GIA), which locally manifests as rebounding of the earth in response to the reduced mass from melting of the Greenland ice sheet (4), and lowering of the local sea surface, due to the corresponding reduction in gravitational attraction (5). Because the magnitude and direction of local sea level change varies around the world, impact studies and adaptation strategies must be locally focused to appropriately represent this predicted variability, alongside variability in local landscapes, and societal factors (6). In-depth knowledge of the local environment, processes, and values is essential to creating useful impact assessments, so studies should genuinely engage local communities throughout the process (7).

The physical processes and parameters that control sea level change vary widely around Greenland and include spatial and temporal variability of the viscoelastic behavior of the solid earth, past and future ice sheet change as well as ocean dynamics and thermal expansion (8, 9). The projections used here are based on the work of Lewright et al. (3) who used an ensemble of GIA models to project sea level to 2100 CE. These models incorporated the response to changes in ice load from three time periods; the end of the Last Glacial Maximum, the Little Ice Age, and future ice changes until 2100 CE. For future ice changes they considered two end-member projections under representative concentration pathways (RCP) 2.6 (low emissions scenario) and RCP 8.5 (high emissions scenario) (10). Lewright et al. (3) also included sea level changes driven by other (non-Greenland) ice mass change, thermal expansion of the ocean, changes in large-scale ocean dynamics, and changes in terrestrial water storage. The total median projected fall in relative sea level at 2100 CE along coastal Greenland can reach a maximum magnitude of 0.9 m for RCP 2.6 and 2.5 m for RCP8.5 (spatial maximum of the ensemble median).

**Fig. 1.**
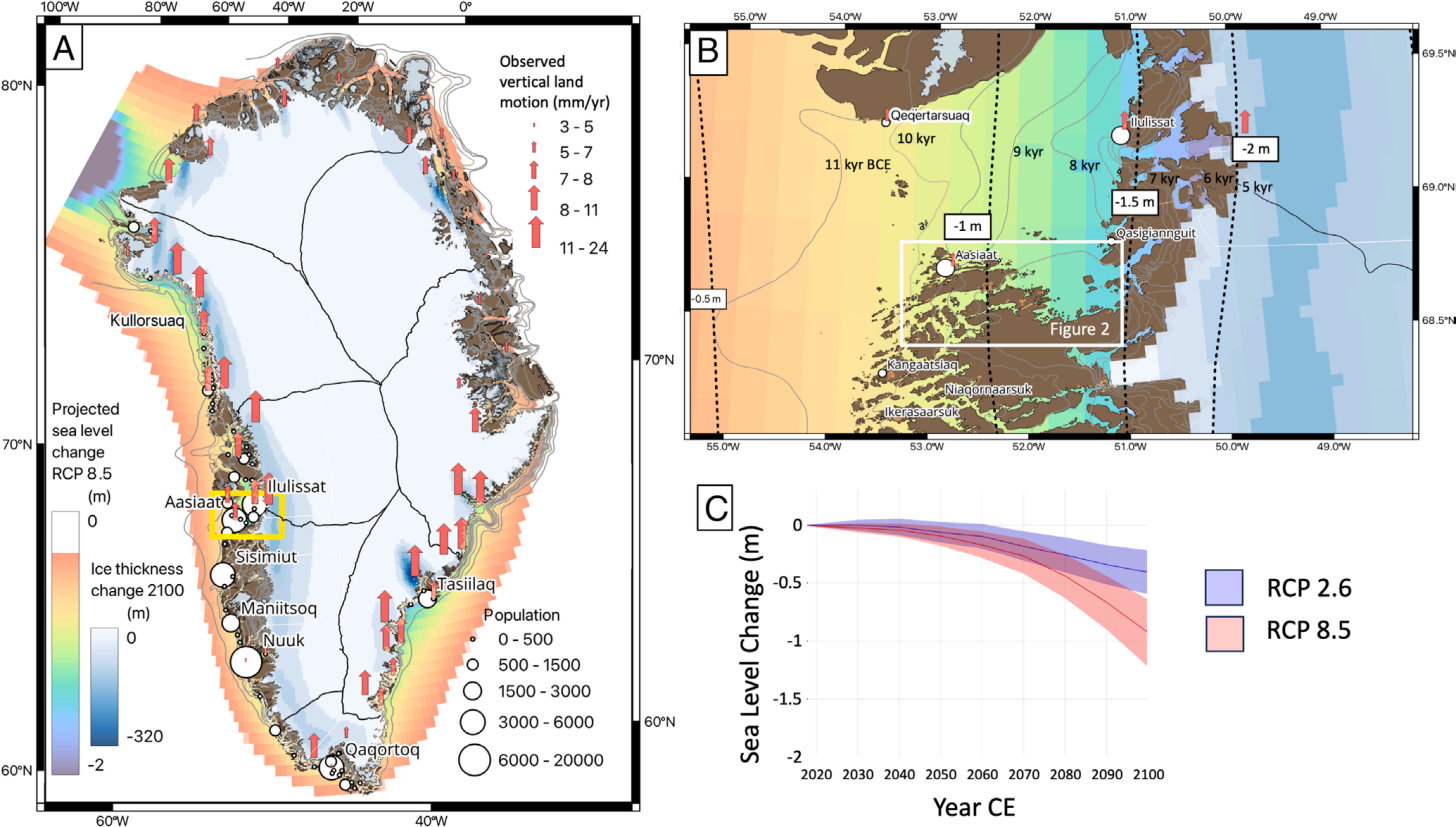
Spatial variability of sea level change and population centers. (*A*) Rainbow colors show sea level projections for 2100, negative values indicate a fall in sea level (3). Blue shading on ice sheet shows mean projected ice thickness loss under RCP8.5 by 2100 (10). White circles indicate population centers, scaled by population size. Red arrows are scaled by observed land uplift rate (4). Black lines show boundaries between major ice catchments, white lines boundaries between municipalities. (*B*) Zoom in on Qeqertarsuup Tunua (Disko Bay) and Aasiaat area. Dotted lines are contours of sea level projections (colored background). Contours offshore show past ice sheet margins since 11,050 BCE (12). (*C*) Projected sea level change for Aasiaat under RCP 2.6 and RCP 8.5 (3).

The impact of changes in sea level on communities depends strongly on individual characteristics of each community. This includes the specific geography of the physical location as well as the human elements of land-use, economy, and daily activities (6, 11). The Greenland Ice Sheet covers 80% of the landmass of Greenland (Fig. 1) and the population is concentrated around the coast with significant day-to-day involvement with the marine environment for commercial activities as well as personal hunting, fishing, travel, and recreation. Future changes in sea level can affect many aspects of daily life, including coastal infrastructure, intertidal habitat distribution, and marine navigation.

Creating relevant predictions for Greenlandic communities requires strong partnerships with Greenlandic people and institutions (13–15). This work, conducted as a partnership between the Greenland Institute of Natural Resources (GINR) based in Nuuk and Columbia University, based in New York, was funded in recognition of the value of bilateral cooperation between US and Greenlandic researchers (16). We focused on developing predictions and assessing impacts of sea level change for communities in coastal Greenland. The project engaged with communities in Nuuk, Aasiaat, and Kullorsuaq in West Greenland and Tasiilaq in East Greenland. Here we focus on the town of Aasiaat to give specific results from one site and illustrate the process that we developed for this investigation.

To assess the impact of reduced water depth we required accurate knowledge of the present-day water depths in areas of importance to the specific community, including very shallow areas where new land could emerge at low tide. Extensive information about the coast and seafloor has been developed by generations of people in Greenland through marine-based travel, fishing, and hunting, resulting in knowledge that cannot be fully represented on maps. Previous scientific mapping of the seafloor has typically been performed far from towns and focused on questions such as resource assessment (17), landscape history, (18) or circulation of ocean water toward the ice sheet grounding line (19, 20). The collaborative design, acquisition, and analysis of bathymetric surveys provided critical quantitative constraints and a common reference for project partners to develop shared understanding of the effects of sea level change in a specific place.

Aasiaat has a population of ~3,000 on the south side of Qeqertarsuup Tunua (Disko Bay) (Fig. 1), part of an archipelago with a large area of shallow sea floor within the Kommune Qeqertalik, the “Municipality of Islands”. Last covered by the ice sheet around 9550 BCE (12), local sea level fell as the land responded to ice retreat. During the Little Ice Age, around 950 CE, a period of ice sheet growth led to local sea level rise (21), similar to that seen elsewhere in Greenland (22). From 2005 CE to 2015 CE, average vertical land motion in Aasiaat of 8 mm/y (4) contributed to falling sea level that is anticipated to accelerate in the coming century (3). Aasiaat contains an ~8 m deep industrial port supporting fishing, offshore exploration, and a growing tourist industry and is a center of education (23). In order to investigate the impacts of local sea level fall in Aasiaat, we identified areas significant to the community and regional biology that could be vulnerable to change and recorded bathymetry and habitats in many of these areas. Combining these bathymetric results with locally targeted projections of sea level change allowed us to assess the anticipated local impacts of sea level fall. We describe the connected range of communication pathways that we engaged to ensure that the survey design was informed and guided by the local community interests and that findings of the project are widely accessible and relevant.

## 1. Results

### 1.1. Collaboratively Designed Bathymetry and Habitat Observations.

#### 1.1.1. Bathymetry.

**Fig. 2.**
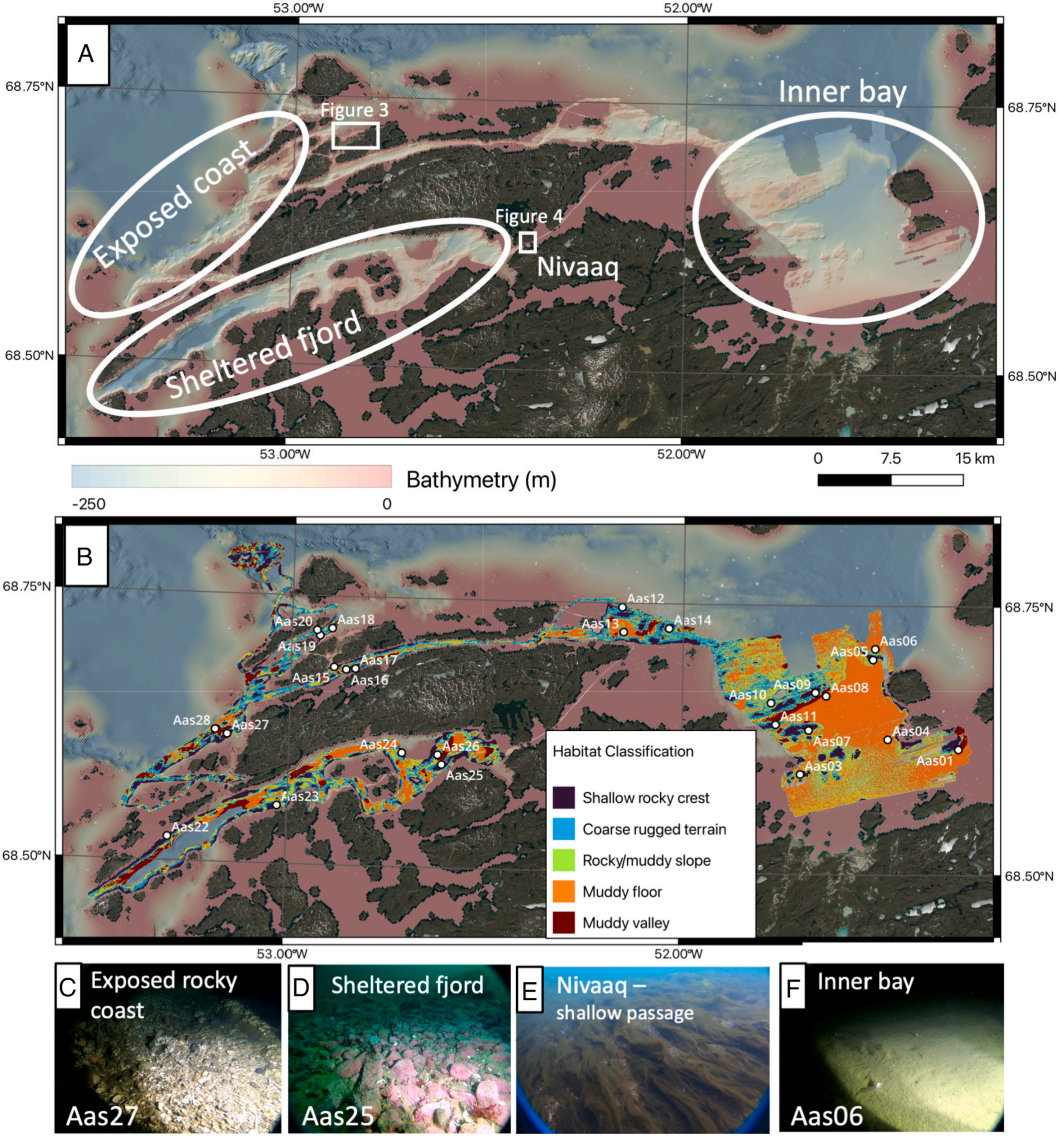
Bathymetry and habitat of Aasiaat area. (*A*) New high-resolution bathymetry surveys highlighted, existing bathymetry (IBSCO) shown as background. (*B*) Habitat classification in color from mid-depth surveys. White dots show location of ground-truth images. (*C*–*F*) Example images illustrating three main regions of survey and the Nivaaq channel.

**Fig. 3.**
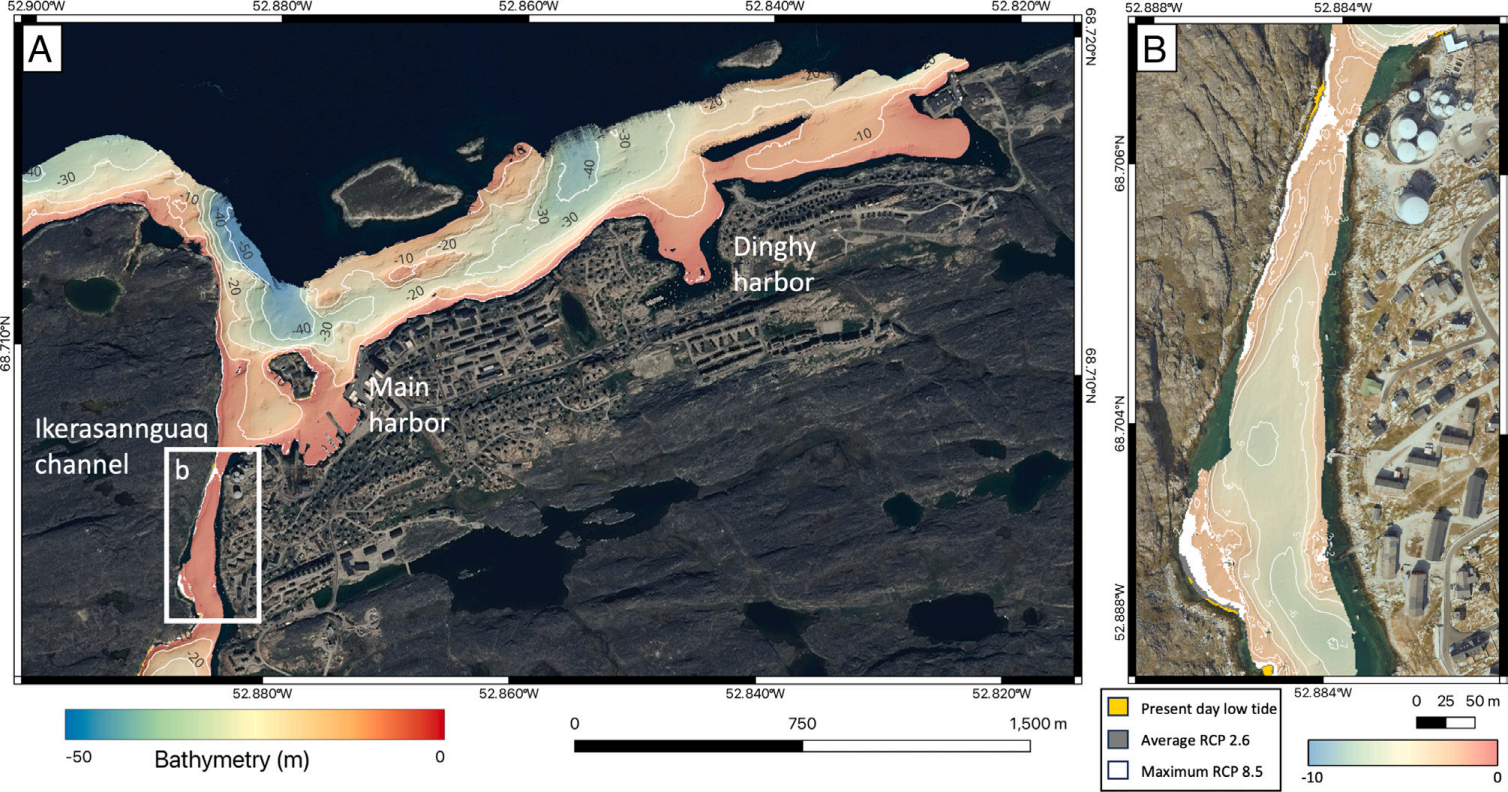
Bathymetry and impact map of the Aasiaat harbor region. (*A*) Bathymetry around the town of Aasiaat with white box showing the location of *B*. (*B*) channel at the western side of the harbor. Impact indicated by filled polygons showing subaerially exposed areas at lowest astronomical tide at present (yellow), and by 2100 under median projections for RCP 2.6 (dark gray) and high (83rd percentile) projected change under RCP 8.5 (white).

**Fig. 4.**
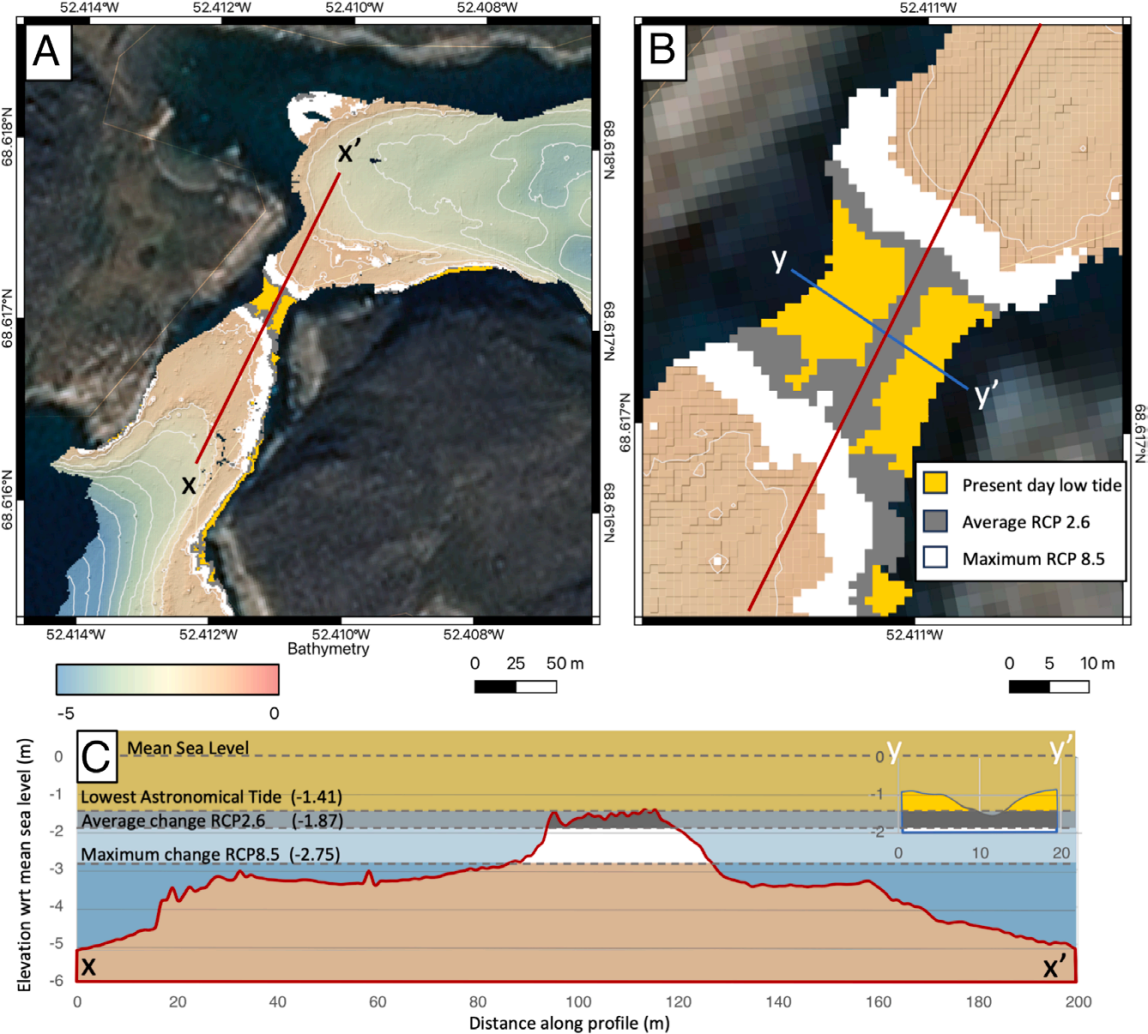
Bathymetry and impact map of the Nivaaq channel region (*A*) and channel (*B*). Impact indicated by filled polygons showing subaerially exposed areas at lowest astronomical tide at present (yellow), and by 2100 under median projections for RCP 2.6 (dark gray) and high (83rd percentile) projected change under RCP 8.5 (white). Red and blue lines indicate location of bathymetric profiles illustrated in (*C*).

High-resolution maps of the seafloor environment quantify the presently submerged landscape and allow comparison with the quantitative projections of future sea level change. The design of these surveys was developed over a series of visits to Aasiaat between July 2020 and June 2021 and included local municipality officials and representatives of hunters and fishers through the organizations KNAPK and PISUNA (*Materials and Methods*). We targeted shallow-water areas where the greatest percentage of water depth change will occur, as well as areas that are significant to the nearby human population, for example harbors and local boating infrastructure, and habitats that are important for hunting or fishing. Bathymetry mapped in the region, and reported with respect to mean sea level (Fig. 2*A*) ranges from −425 m at the deepest point to areas that are so shallow they could only be surveyed at high tide. We monitored the tidal range with a tide gauge installed at Aasiaat harbor from July 2021 to September 2022 and recorded a ~3 m average range. Near Aasiaat harbor, water depths are seldom greater than −50 m, with depths less than ~−2 to −3 m near the shore, including the small dinghy harbor, the main harbor, and the Ikerasannguaq channel to the west of the town (Fig. 3*A*). Additional shallow regions were mapped along the shoreline at Akuunaq, and in the Nivaaq channel. The Nivaaq channel is 40 m wide with an average depth of ~1 m below mean sea level (Fig. 4*A*). A central passage, 3 m wide and −1.6 m deep, cuts through the full 25 m length of the shallowest part of the Nivaaq channel. Elsewhere water depths typically extend to ~−220 m, with isolated regions below −300 m.

#### 1.1.2. Habitat and ecology.

We developed habitat maps for the deeper water by combining physical measurements of the seafloor environment with ground truth photographs and video. The benthic habitats are defined based on seafloor depth, slope, and backscatter reflectivity, an indication of the acoustic hardness of the sea bed (24), and further validated with ground-truthing. The deepest categorized habitat ranges from −160 m to −220 m depth and is muddy (low backscatter intensity of −46 to −57 dB), and flat (i.e. <5 degree slopes) or has local depressions. At medium-shallow depths (−100 to −125 m) and areas of steep slopes (20 to 35 degrees), we see bedrock with a mixture of mud, boulders, and pebbles. Shallow areas occurring between −50 to −100 m are characterized by moderate slopes (5 to 20 degrees), some crests, and mixtures of rock and mud with backscatter ranging from −27 to −31 dB. The resulting habitat classification map (Fig. 2*B*) extrapolates ground truth observations to characterize the survey area according to six habitat classifications: Shallow rocky crests; Rocky slope, Rocky/muddy slope; Coarse rugged terrain; Muddy floor; Muddy valley.

The eastern side of the survey area, in inner Qeqertarsuup Tunua, is characterized by an extensive muddy plain showing iceberg plow marks and pits around its margins (Fig. 2*F*) and more complex habitats closer to shore. The western edge of the survey area includes the exposed outer coast that faces toward Davis Strait (Fig. 2*C*). This area was mostly bedrock or coarse rocky ground with occasional muddy patches. The inner bay and outer coast are connected through an inner fjord system that includes the narrow Nivaaq channel (Fig. 2*E*). The eastern end of this system was a mix of gravel and bedrock. Close to the Nivaaq passage, shallow coarse rocky areas progressed to mud flats at depth.

Within the shallow channel Nivaaq, satellite imagery shows dark material covering the seafloor. Ground truth video confirms the presence of macroalgae with dense aggregations of sea urchins and tightly packed beds of blue mussels (Fig. 2*E*). Vigorous water flow and a large number of mussels were visible, demonstrating active feeding in the narrow passage. Deep, muddy seafloor to the west of Nivaaq (−200 m, site 26) reflects deposition in areas of very slow flow. At shallower depths (−50 m, site 25) encrusting of red algae on rock shows that sediment deposition is not occurring.

### 1.2. Locally Targeted Predictions of Sea Level Change.

#### 1.2.1. Impact mapping.

We merged the new bathymetric results with the projections of sea level at 2100 CE to identify areas where local sea level fall would have an impact on the community and ecosystems. Projections for emission scenarios RCP 2.6 and RCP 8.5 show that the Aasiaat region is expected to experience a fall in sea level of between 0.41 (17th to 83rd percentile range 0.58 to 0.22) m and 0.93 (17th to 83rd percentile range 1.22 to 0.64) m by 2100 CE (3). These estimates include GIA in response to past and future load changes (ice and ocean), changes in ice sheet and mountain glaciers, changes in terrestrial water storage, thermal expansion, and changes in large-scale ocean dynamics. Impacts will be greatest in shallow water where change is a significant proportion of water depth. We identified highly vulnerable regions by adding predictions of sea level change to present-day water depths measured to the datum of Lowest Astronomical Tide (1.41 m below mean sea level) to define areas predicted to be newly subaerially exposed at low tide by 2100 CE. The low-change scenario considers the median projection for RCP2.6 to represent the most likely low-change case while the high change scenario considers the high-end (83rd percentile) projected change for RCP 8.5 in order to illustrate a less-likely but possible high-end scenario that is relevant to the risk considered by coastal planners (25). We do not account for changes to tide elevations by 2100 CE, which can be large and highly variable, depending on local configurations of coastline and substrate (26, 27). This is discussed further in Section 2.3.2. The highlighted areas (Figs. 3 and 4) will become subaerially exposed by 2100 CE. These areas are typically seaward extensions of the present-day coast, but in some places newly exposed seafloor appears mid-channel, or connects across the entire channel width of narrow channels. For example at Ikerasannguaq, to the west of Aasiaat harbor, the channel would close (Fig. 3) under the most extreme scenario for RCP 8.5, while Nivaaq will be closed under any illustrated scenario (Fig. 4). This tangible change—newly emergent land as the land rebounds and sea level falls—can be easily communicated to a wide audience.

#### 1.2.2. Sensitivity to local and regional ice mass change.

A significant contribution to the uncertainty in the sea level projections used here is the range of plausible future ice sheet evolutions, which is large even for a given RCP scenario (Fig. 1*C*). To understand where ice sheet processes and model accuracy most matter for local sea level change in Aasiaat we quantified how sea level at Aasiaat depends on ice sheet evolution across the globe by calculating an ice sensitivity kernel using the adjoint method (*SI Appendix*, Fig. S1 *A*–*C*; see *Materials and Methods*). Using the sensitivity kernel we then estimated each ice catchment’s contribution to sea level change at Aasiaat (Eq. **1**).

In these projections, Aasiaat’s sea level is dominated by ice loss in the west and south west of Greenland. For example, ice loss from the ice sheet basin drained by Sermeq Kujalleq [formerly known as Jakobshavn Isbrae (28)] causes a fall of sea level in Aasiaat (28 cm) that is six times greater than the amount of sea level fall contributed by the basin on the east coast drained by Helheim Gletsjer (4.7 cm), and 25 times more than that drained by Kangerlussuaq Gletsjer (1.1 cm) by 2100 under RCP 8.5 (*SI Appendix*, Fig. S1*D*). In contrast, if GIA processes were ignored and sea level was assumed to change uniformly these ice basins would raise sea level by 1.0, 0.9, and 0.5 cm, respectively. Because the mean RCP 2.6 ice sheet evolution is spatially similar to that of RCP 8.5 and because the ice sensitivity kernel over this short duration is insensitive to the differences between these two ice sheet evolutions the statements above remain true though the magnitudes of sea level change are smaller (*SI Appendix*, Fig. S1*E*).

### 1.3. Communication Pathways and Data Products.

#### 1.3.1. Communication pathways.

Collaboratively designed multibeam mapping of the seafloor acted in this project as an interface and translator between the extensive local knowledge of the different systems influenced by changes in the marine environment, and the quantitative projections of future change provided by models. Ongoing communication through several pathways was an essential element of the development and distribution of new findings. New communication pathways were established throughout this work, including media outreach, institutional meetings, interpersonal meetings, and community engagement.

Early engagement through media outlets reached a broad audience to convey key concepts of sea level change near melting ice sheets, and the questions of coastal impacts. Reports in a Greenlandic National newspaper, radio, and television were combined with public presentations at events such as Nuuk Culture Night to allow reciprocal communication with attendees.

Existing institutional relationships between the Greenland Institute of Natural Resources and the Naalakkersuisut (Government of Greenland) enabled ongoing contact with the Ministries of Infrastructure and Education, the regional municipality administration for Aasiaat Kommune Qeqertalik, and other municipalities Avannaata Kommunia, and Kommuneqarfik Sermersooq. These pathways provided insight on current governmental concerns and future plans.

Initial engagement with the focus communities was through existing contacts and direct communication with community groups through social media. Recurring in-person visits in each year of the project (2020–2024) developed trust through extensive personal interactions between community members and project personnel. Informal meetings and community gatherings facilitated survey design and developed pathways for sharing results.

Classroom activities and engagement with formal education supported capacity building within the Greenlandic education system and fostered exchange between students and role models. Key concepts including sea level change near ice sheets, tides, multibeam mapping, ecosystem assessments, benthic habitats, and science careers were developed into classroom materials in Kalaallisut (Greenlandic), Danish, and English. Team members delivered these materials to student groups in Aasiaat’s upper elementary school and high school. Emphasizing the importance of local knowledge and the intergenerational timescales of earth processes, students were encouraged to interview family members to understand the changes they had observed over their lifetimes. Employment opportunities in scientific fields within Greenland were articulated through sharing and discussing the Greenlandic team members’ career paths.

#### 1.3.2. Sharing data products.

Developing useful data products relied on strong reciprocal communication pathways to provide accessible and relevant results to multiple interested groups. The bathymetry and habitat maps developed here have broad-reaching relevance for scientific, policy and community-based interests. These products were provided to government agencies and data centers including the Danish Hydrographic Office and the Arctic Data Center as well as a written report on the conception, methodology, and outcome directly to the Qeqertalik municipality and the Ministry of Housing and Infrastructure of Naalakkersuisut. We publicly distributed preliminary bathymetry data soon after collection in the form of printed maps and through a commonly used website (Avenza) that allows users access georeferenced maps on their mobile devices. Updateable digital resources summarize the overall project through ESRI StoryMaps narrated in Kalaallisut, Danish, and English. The StoryMaps include web maps that give access to the ground truth benthic photographs in context of seafloor bathymetry. Hard-copy poster summaries of the project in each language were hung in public spaces, with QR-code links to all project products ranging from the bathymetric maps to the educational material. By sharing fundamental data on platforms tailored to the local community, products were immediately and enduringly available for application beyond the project focus.

## 2. Discussion

### 2.1. Predicted Impacts.

#### 2.1.1. Nivaaq navigation and ecosystem shifts.

As bedrock uplifts and sea level drops more land will be exposed at low tide, effectively closing the Nivaaq channel with wide-ranging effects for small-craft navigation and ecosystems (Fig. 4). Currently, at high tide the Nivaaq channel provides a safe inshore route between the small coastal towns to the west with the commercial hubs of Aasiaat and Ilulissat. As it becomes shallower more vessels will be forced to take longer, more exposed offshore routes.

The benthic ecosystems imaged at Nivaaq showed evidence of high productivity and fast water flow through the narrow, shallow channel during maximum tidal flow (Fig. 2*E*). Large clusters of blue mussels observed at Nivaaq provide habitat for large populations of invertebrate fauna that could sustain additional populations in other parts of the fjord. A dramatic reduction or loss of this habitat as the channel becomes shallower or completely closes might affect invertebrate populations over a broad region through a decrease in new recruits or a disruption to recruitment pathways (29). Determining the broad-scale ecological impact of these channels closing is a complex topic of further study, but it is clear that closure will cause local disruption and potentially collapse of site-specific populations, with consequences beyond the immediate area.

#### 2.1.2. Harbor area and infrastructure.

Shoaling waters will also reduce the utility of the present-day harbors. Deep draft boats will find harbors inaccessible, while strong currents in shoaling channels will become more hazardous. Access to current mooring sites will become increasingly difficult in shallow areas as the shoreline moves seaward. In Aasiaat, predictions show newly emergent islands through the Ikerasannguaq channel on the west side of the archipelago (Fig. 3 *A* and *B*), and within the small dinghy harbor, hindering navigation and increasing the challenge of accessing moored boats during inclement weather (Fig. 3*A*). The mapped impact areas are conservative as the harbor will become unusable before it becomes subaerially exposed. The changes in the dinghy harbor and the Ikerasannguaq channel are particularly important to consider as the municipality plans new harbor infrastructure. Site-specific projections enable the specifications and location of new harbors to be designed to accommodate the projected changes in sea level over the life of the structure. Possible responses to shallowing water include artificially deepening existing areas and channels by underwater blasting or dredging, or using alternative navigation routes.

#### 2.1.3. Threshold response.

The shoaling and narrowing channels at Ikerasannguaq and Nivaaq highlight the importance of critical depth thresholds, where steady change in sea level can result in step changes in impacts when critical depth thresholds are reached. The combination of GIA and ocean processes are projected to cause accelerating sea level fall that averages 6 to 13 mm/y from 2025 CE to 2100 CE. The effect of steady changes can be incorporated into planning of any future infrastructure by taking into account the rate of change and how much time the infrastructure is expected to endure to maintain functionality. Step changes in functionality of waterways may require different planning strategies and interventions. The targeted mapping performed here highlights critical depths at which step-changes in navigation conditions can occur, for example when the Ikerasannguaq channel will first become impassable at low tide. Shallow water ecosystems can also be expected to adapt to a slowly changing environment, but are vulnerable to key depth thresholds, such as when flow through the channel is entirely cut off, causing a regional circulation reorganization. Future work is needed to assess the full scale of the impact of predicted channel closures, and whether local engineering projects are desired to counteract the impacts of global change.

### 2.2. Impact of Integrated Communications.

The ongoing engagement between science personnel and community experts that was critical to developing bathymetry data and impact predictions also resulted in impacts beyond the scope of the original investigation.

The approach to travel and engagement was influenced by the worldwide COVID19 pandemic, which led to restrictions on public gatherings and international travel from 2020–2021, and changes in approach to a wide range of scientific projects in polar regions (30). Initial meetings were postponed, and then revised from the planned town-hall format to small, one-on-one meetings attended only by Greenland-based personnel. International travel to communities was avoided for two years after restrictions were emplaced, with the first postpandemic international visits to communities occurring in 2022. Some of these changes in approach produced positive outcomes, for example project-focused curriculum activities designed to present STEM materials locally relevant to students, and to inspire further engagement in science within Greenland, were first delivered by Greenland-based team members. This strengthened both their connection to Greenlandic learning styles and their impact with the students. Additionally, survey work that would have been performed by members of the US team was instead done by Greenland-based masters students supervised by the Greenland science team, providing them with unanticipated training opportunities. Contributions from students ranged from sharing their own knowledge and observations of the coastal environment, helping to acquire new bathymetric datasets and contributing to the interpretation of results in Kalaallisut, demonstrating the reciprocal value of local student participation.

Community members’ central role in the data collection enhanced their investment in project results. Additionally, the broad interest by community members led to new considerations in developing resources that would be relevant for delivering results to the varied audiences. For example the bathymetry maps on Avenza have been downloaded hundreds of times. Final products were accepted by the Danish Hydrographic Office as fit for use in future navigational charts and by local and national government offices, where they can be taken into consideration in planning future harbor developments.

In support of ongoing work on future regional sea level and local water level changes, Oqaasileriffik, the Language Secretariat of Greenland, recognized the need for new terms in Kalaallisut. In 2024, Oqaasileriffik authorized the development of new terms in Kalaallisut for referring to local, rather than global water level. The term *immap killinga* has been adopted to describe water level in a regional environmental, geodetic, and oceanographic context (31) while *ermup qaffasissusia* describes water level on a local and temporal scale (32), enabling precise discussion of sea level change among Kalaallisut speakers.

The multiple-phased investigation required annual visits and considerable investment of time from both science personnel and community-based participants. The relationships developed during this time facilitated new contacts in other communities, supporting the broader objectives of the Greenland Rising project as well as creating known points of contact for future investigations.

This style of engagement is time consuming for community members as well as science personnel, and is not recommended for every investigation. In the case of this project, engagement of Greenland-based project members was critical to the reciprocal communication between local experts and international investigators, but was entered into with care and invitation from Greenlandic communities and organizations. Creating opportunities for participation from Greenlandic students was an effective and mutually beneficial way of completing key objectives of this study. The organization Arctic Hub, established in 2020 is an example of investment in the principles articulated in the Greenland National Research Strategy (15), to help facilitate mutually advantageous relationships between the Greenlandic population and international researchers.

### 2.3. Direction for Future Work.

#### 2.3.1. Reducing uncertainty from ice sheet projections.

Projected change in future sea level increases significantly with proximity to the ice sheet, with up to 1 m variation across Qeqertarsuup Tunua (Fig. 1*B*) with an extra 0.5 m of sea level fall expected at Ilulissat compared with Aasiaat. As well as highlighting the importance of local projections of sea level change, these results can be used to identify other locations where the impact of sea level change around Greenland will be significant.

The sensitivity kernel indicates that for a given unit of mass change per area, the evolution of the nearest two ice basins have an order of magnitude stronger impact on sea level change at Aasiaat than all other glaciated regions in the world (Fig. 1). Over longer timescales than those examined here, the volume of potential ice loss from the Antarctic ice sheets, mountain glaciers, and sea level rise due to thermal expansion could far exceed the contribution to sea level change from local basins. However, over the century-scale timeframe, uncertainties can be reduced by improved understanding of glaciers within the two local catchments, which include the major ice stream Sermeq Kujalleq, where uncertainties in ocean forcing cause large variations in the projected future mass change (10).

#### 2.3.2. Additional processes to be considered.

The sea level projections used here consider a range of different GIA models, incorporating different treatment of mantle properties, modeled past and future ice volumes, and changes to sterodynamic ocean properties. Future sea level and its influence on the lived environment can also be affected by changes in tides, storm patterns and wave action as well as geomorphological processes of erosion, sedimentation, and permafrost change. These processes are not considered here. Sea level change can cause changes in tides through changes in water depth, basin shape and basal friction, and shallow-water regions can therefore be particularly sensitive (26). The study presented here provides critical information to support future understanding by accurately constraining the bathymetry within the detailed coastal system of the Aasiaat fjords as well as installing a new tide gauge within Aasiaat harbor. We have highlighted areas along the generally steep, rocky coasts of Aasiaat where lateral shorelines might show significant change as well as mapping the seafloor substrate of intertidal regions within the Nivaaq channel where basal friction might change with changing ecology. Tide gauge measurements remain sparse across the Arctic, and additional tide gauge observations at key sites would improve predictions of future impacts, and with a sufficiently long time series would allow direct observation of sea level change. Integrating GNSS-IR based sea level observations into the tide gauge network (33) and augmenting these with satellite observations (34, 35) might be promising directions. Furthermore, studies of changes in tidal variation over this century will be important for understanding and quantifying impact.

While sedimentation and erosion can also modify local sea level impacts, the absence of major sediment outlets in the Aasiaat survey area, and the muddy floor of the submarine channels, suggest low sedimentation rates in relatively still environments. Along the coast, widespread exposed bedrock indicates low coastal erosion rates. In areas closer to major outlet glaciers, with high sediment loads and greater projected sea level fall, sedimentation can be expected to be relevant. Although not of active concern in Aasiaat itself (36), the region around Aasiaat has a high potential for permafrost thaw which can lead to land subsidence counteracting the regional sea level fall.

## 3. Conclusions

In this work we have developed locally relevant predictions of the impacts of sea level change by combining location-specific projections from global models of glacial isostatic adjustment with new, collaboratively developed observations of seafloor bathymetry. Each stage of the project depended on reciprocal communication between science personnel and local experts.

While steady changes in sea level can usually be ecologically accommodated by a range shift of benthic species, threshold depths were identified within narrow channels at Nivaaq and Ikerasannguaq which would result in disruption of circulation of water and nutrients between basins, with wide-ranging ecological impacts. A fall in sea level of between 0.41 (17th to 83rd percentile range 0.58 to 0.22) m and 0.93 (17th to 83rd percentile range 1.22 to 0.64) m is projected for Aasiaat by 2100 CE, for RCP 2.6 and 8.5, respectively. The new seafloor mapping quantified the depth of the seafloor around Aasiaat harbor and in key passageways that are important for marine navigation and identified critical areas that could be newly subaerially exposed at lowest astronomical tide by 2100 CE. Predictions showed reduced access through the Ikerasannguaq and Nivaaq channels as well as the shallow dinghy harbor used for mooring. Newly emergent rocks currently at or near sea level will make navigation more hazardous.

Broad engagement of the community was central to understanding ongoing change and design of the impact assessment. We employed media outreach, government official briefings, community meetings, individual interviews, formal education and information outreach programs in the design, acquisition, and interpretation of seafloor surveys. The data and results are available beyond the scope of the original project goals through a series of multilingual materials, including digitally downloadable and interactive maps, classroom materials, physical models, and hard-copy posters. Local scientific leads played a critical role in developing and maintaining reciprocal communication across three languages, including the adoption of new terms into Kalaallisut. Local student participation provided valuable contributions to the key goals of the project as well as training opportunities. By using the development of baseline datasets for capacity building within science education in Greenland this project laid groundwork for deeper understanding of the interrelated processes of sea level change and their impacts on Greenlandic community life.

High gradients in sea level projections around Greenland highlight the value of extending this work to other coastal communities. The approach used here can also be adapted to communities in other environments where a focus on community engagement, shared observations, and relationships with local experts allows global-scale models to support locally relevant decision-making.

## 4. Materials and Methods

### 4.1. Community-Focused Survey Design.

To design relevant bathymetric surveys, Greenland-based project personnel visited the community of Aasiaat in July 2020 to meet with community members and identify priority survey targets. As this visit was during the COVID-19 pandemic, which limited travel and public gatherings, meetings were held with small groups of community representatives. The project was widely publicized in Greenlandic media prior to the visits, with an article in the national newspaper Sermitsiaq (in Kalaallissut and Danish), posts to community Facebook groups and a national television interview on Kalaallit Nunaata Radioa (KNR). During the visit to Aasiaat, team members appeared on the radio to introduce themselves and the project. Meetings were arranged with local municipality officials, and representatives from PISUNA and KNAPK.

Meetings focused on discussion about how the coastal environment is currently changing, and where future changes might be expected to have impact. According to the preference of the participants, meetings were conducted in Kalaallissut or Danish with maps of each area being annotated through the conversation to locate key features of interest. Team members also delivered curriculum materials in schools introducing the project concepts, drawing attention to the importance of the local coastline and encouraging students to talk with their families to learn about observed changes on the coast.

A summary of the annotated maps was presented to the science team after the visit. High priority survey areas, with great significance to the community and likely to experience a high percentage change in sea level, were agreed on. Highest-priority areas included the industrial and dinghy harbors and shallow seaways including the Ikerasannguaq channel to the west of Aasiaat town, and the narrow, shallow passage at Nivaaq. Additional areas include deeper water fishing and hunting areas that were less vulnerable to being subaerially exposed, but may be affected by broad-reaching changes in the environment, such as cascading impacts from the present-day tidal zone ecosystems, or changes to sea state or sediment supply from regional sea level changes.

### 4.2. Multibeam Bathymetry.

Mid-depth (~−50 to −800 m) waters were mapped from the GINR vessel RV Sanna between 27 July and 6 August 2020. Shallow-water mapping (~−2 to −150 m) was conducted from a charter vessel from 7 to 15 June 2021. Sanna surveys used a Teledyne RESON Seabat T50-R ER multibeam sonar system to measure depths and backscatter data. Data from the sonar were geographically referenced using high-precision GNSS and Inertial Motion Unit instruments. Water column sound velocity necessary to adjust multibeam measurements was sampled with a Valeport sound velocity probe (SVP). In the shallow waters, the chartered small boat ANGAATUAQ, a Finnmaster Pilot 7, was outfitted with a Norbit WMBS multibeam system, Applanix POS MV Surfmaster II motion sensor and two Trimble GNSS antennas for position and heading. Sound velocity values were derived from an online probe attached to sonar and a small Valeport mini SVP deployed manually each day. Full resolution data resolve features on a scale of 0.5 to 5.0 m dependent on depth ranges. After individual swaths were leveled to adjacent survey areas to create a cohesive grid, the estimated uncertainty of the final grid is ~0.2 m.

### 4.3. Habitat Classification.

A combination of seafloor depth and backscatter was used to classify morphology using Benthic Terrain Modeler toolbox in ArcGIS (37). These morphology classifications were validated with digital ground-truthing from drop camera and towed video footage. Based on the acoustic measurements and the ground-truth the final “habitat classes” follow the Greenland Ocean floor Classification of Habitats (38).

### 4.4. Tide Gauge.

Tide gauges were installed for both geodetic and education/outreach purposes. An RBR Duo T.D tide logger was installed within a 6.5 m plastic pipe, secured to the side of the harbor wall in July 2021 and surveyed in position with a Leica GNSS receiver. Eighteen months of data were recovered from the instrument in September 2022, when it was removed and reinstalled.

Due to the scarcity of tidal records around Greenland, we have applied tidal constraints from Aasiaat harbor across the entire survey area. To assess the impact of this simplification, we ran an astronomical tide model PyTMD (39) to compare predicted variation between sites. The 2% change (6 cm) between the Aasiaat and at Nivaaq modeled tidal ranges is well within the ~20 cm accuracy of the multibeam survey and supports the application of uniform correction to the LAT datum.

### 4.5. Impact Predictions.

In order to identify key areas that are vulnerable to future sea level change we used a simple first-order model to map areas that would be newly exposed at lowest astronomical tide. Bathymetry data, initially processed to the Greenland GEOID16 were shifted to a datum of lowest astronomical tide (LAT) using offsets from DMI tide gauge records (40). Depths below this datum are not currently subaerially exposed at low tide. We then added projected changes from Lewright et al. (3) to the bathymetry to examine the impacts of future sea level fall. For a low-change scenario we used the median projected sea level change for RCP 2.6 and for the high change we used the 83rd percentile projected change under RCP 8.5. Areas with projected elevations above the LAT datum were highlighted on the resulting impact maps. By treating the future sea level projections as a change in water column depth we combine the effects of land uplift and lowering of sea surface.

### 4.6. Spatially Variable Sensitivity.

The ratio of the sea level change at an observation site with respect to the rate of change of ice thickness at an arbitrary point in space and time, $\partial SL/\partial \dot{I}$, is a partial derivative or Fréchet derivative and its full geographic mapping across Earth’s surface and through time, t, is an ice sensitivity kernel, $K_{\dot{I}}$ (e.g., *SI Appendix*, Fig. S1 *A*−*C*). The magnitude of $K_{\dot{I}}$ at any given point in space and time determines how strongly sea level at the observation site (e.g., Aasiaat) will change given a change to the ice sheet evolution, $\dot{I}$, while the polarity of the kernel will determine whether the perturbation to the ice sheet evolution causes sea level to rise or fall at the observation site.

We use the adjoint method to calculate the ice sensitivity kernel, $K_{\dot{I}}$, for sea level at Aasiaat at 2100 CE by performing two numerical simulations: 1) a forward simulation driven by the ice sheet evolution, which in this unique case is held fixed, and 2) an adjoint simulation driven by a “fictitious” adjoint sea level load at Aasiaat. Both simulations are performed at spherical harmonic degree 256, adopt the elastic and density structure of PREM (41), and a 3D viscosity inference (42) based on the shear wave speeds of GLAD-M25 (43). Using the state variables from both the forward and adjoint simulations we calculate the ice sensitivity kernel following equation 104 in (44) (*SI Appendix*, Fig. S1 *A*–*C*).

Given the ice sensitivity kernel, $K_{\dot{I}}$, for Aasiaat we can estimate to first order how sea level at 2100 CE will change given a perturbation to the ice sheet evolution, $\dot{I}$, by solving

[1] $$\delta SL=\int_{2017}^{2100}\int_{\partial M}K_{i}\delta \dot{I}\;dS\;dt,$$

in which the surface integral over ∂M can be rapidly evaluated using spherical harmonic transforms and the time integral is evaluated using the trapezoid rule. Within this simple expression, the sensitivity kernel captures the influence of all physical processes of the system to first order, which in our example includes viscoelastic deformation due to changes in ice and ocean loads on a spherical Earth with a 3D mantle and lithosphere, self-gravitation, and shoreline migration. We evaluate Eq. **1** eighteen times to estimate the contribution of sea level change at Aasiaat by 2100 CE for each of the major Greenland ice basins and Antarctica given mean ice sheet evolutions associated with RCP 2.6 and 8.5 (*SI Appendix*, Fig. S1 *D* and *E*). The full set of calculations based on Eq. (**1**) require less than a minute to complete and thus, are significantly faster than the ~4 h required to solve our small forward GIA problem once or ~3 d to complete 18 forward GIA simulations to determine the contribution to Aasiaat sea level from each region.

## Supplementary Material

Appendix 01 (PDF)

## Data Availability

Bathymetry data are available from the Greenland Rising portal at the Arctic Data Center (45).
