# Supplementary material for: Impacts of future sea level change on Greenland from community knowledge, coastal mapping, and glacial isostatic adjustment models
Source: Proc Natl Acad Sci U S A. 2026 Jun 1;123(23):e2528615123. doi: 10.1073/pnas.2528615123 (PMC13250546; doi:10.1073/pnas.2528615123)
Supplement: Supplementary file 1 — Appendix 01 (PDF) [file pnas.2528615123.sapp.pdf]

Supporting Information for

Impacts of future sea-level change on Greenland from community knowledge, coastal mapping and glacial isostatic adjustment models

Kirsty J. Tinto, Jacqueline Austermann, Robin E. Bell, David Blockley, Casey E. Brayton, Diana Krawczyk, Lauren Lewright, Andrew J. Lloyd, Frank O. Nitsche, Guy J.G. Paxman, David F. Porter, Aqqaluk Sørensen, Margie Turrin, Karl Zinglersen

Kirsty J. Tinto Email: tinto@ldeo.columbia.edu

This PDF file includes:

Figure S1

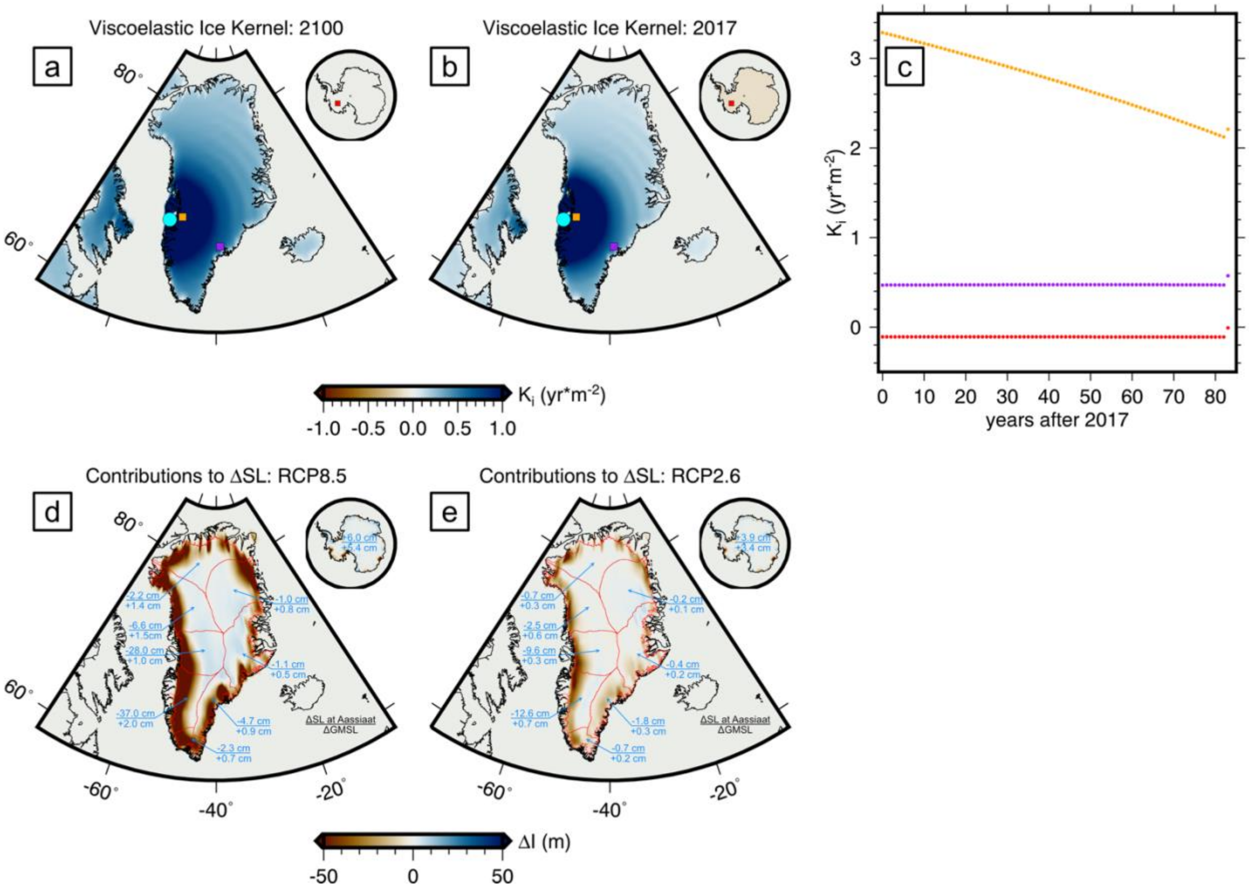

Supplementary Figure 1. Sensitivity of Sea Level to the rate of ice thickness change, $\dot{I}$, and an example of estimating ice basin attribution to 2100 sea level change at Aasiaat (Cyan circle). Here sea level change reflects the time period 2017-2100. (a,b) Map view of the "ice" sensitivity kernel at 2100 and 2017 in Greenland and Antarctica (inset). Positive (negative) kernel values indicate that an increase in the rate of ice thickness change (i.e., mass gain) will cause an increase (decrease) in sea level at Aasiaat. (c) Ice sensitivity kernel as a function of simulation length for 3 locations (Sermeq Kujalleq – orange, Helheim Gletsjer – purple, West Antarctica - red), which are shown as colored squares in (a) & (b). The amplitude of the orange curve increases towards 2017 (i.e., 0 years) because the viscous response due to load changes at the orange square strongly impacts sea level at Aasiaat. In contrast, no significant change in the purple or red curves are observed over the 84-year period because of their greater distance from Aasiaat. This is because during this short time, load changes at these locations (purple and red) cause no meaningful vertical solid Earth deformation at Aasiaat that would further modify its sea level. (d, e) Shows the mean ice thickness change[1] from 2017 to 2100 for RCP 8.5 and RCP 2.6. The red lines demarcate the major Greenland ice basins[2] and the number pairs for each basin report its contribution to 2100 (top) regional sea level change at Aasiaat due to GIA and (bottom) the global mean sea change (i.e., no GIA) estimated from equation 1 and the ice sensitivity kernel shown in (a), (b), and (c).

[1] Goelzer, H. *et al.* The future sea-level contribution of the Greenland ice sheet: a multi-model ensemble study of ISMIP6. *The Cryosphere* **14**, 3071–3096 (2020).

[2] Ekholm, S. A full coverage, high-resolution, topographic model of Greenland computed from a variety of digital elevation data. *Journal of Geophysical Research: Solid Earth* **101**, 21961–21972 (1996).
